# The molecular mechanism for activating IgA production by *Pediococcus acidilactici* K15 and the clinical impact in a randomized trial

**DOI:** 10.1038/s41598-018-23404-4

**Published:** 2018-03-22

**Authors:** Tadaomi Kawashima, Naho Ikari, Tomoko Kouchi, Yasuyuki Kowatari, Yoshiro Kubota, Naoki Shimojo, Noriko M. Tsuji

**Affiliations:** 1 Research and Development Division, Kikkoman Corporation, Chiba, Japan; 2 Biomedical Research Institute, National Institute for Advanced Industrial Science and Technology (AIST), Tsukuba, Japan; 3 Ueno Clinic, Aisei Hospital, Tokyo, Japan; 4 Kikkoman General Hospital, Kikkoman Corporation, Chiba, Japan; 5 Department of Pediatrics, Graduate School of Medicine, Chiba University, Chiba, Japan

## Abstract

IgA secretion at mucosal sites is important for host defence against pathogens as well as maintaining the symbiosis with microorganisms present in the small intestine that affect IgA production. In the present study, we tested the ability of 5 strains of lactic acid bacteria stimulating IgA production, being *Pediococcus acidilactici* K15 selected as the most effective on inducing this protective immunoglobulin. We found that this response was mainly induced via IL-10, as efficiently as IL-6, secreted by K15-stimulated dendritic cells. Furthermore, bacterial RNA was largely responsible for the induction of these cytokines; double-stranded RNA was a major causative molecule for IL-6 production whereas single-stranded RNA was critical factor for IL-10 production. In a randomized, double-blind, placebo-controlled clinical trial, ingestion of K15 significantly increased the secretory IgA (sIgA) concentration in saliva compared with the basal level observed before this intervention. These results indicate that functional lactic acid bacteria induce IL-6 and IL-10 production by dendritic cells, which contribute to upregulating the sIgA concentration at mucosal sites in humans.

## Introduction

A variety of lactic acid bacteria (LAB) from fermented foods or human microbiota show multiple beneficial effects on human health^1–4^. Recent studies revealed that probiotic strains of LAB activate the innate immune system and then activate the acquired immune system, resulting in the prevention of immune diseases^5–9^ and protection against bacterial and viral infection^10–13^. Firstly, LAB stimulate the immune system via the induction of type I interferons (IFNs)^14^, which play an important role in anti-viral immune responses^12,13^. The production of type I interferons (IFNs), particularly IFN-α and IFN-β from dendritic cells (DCs) upon LAB-stimulation were reported beneficial for exerting an anti-viral effect against influenza virus^5,15,16^.

Another major mechanism of LAB to improve host defence in the gut is enhancement of production in specific antibodies (Ab) against pathogens, and the overall increase in total IgA^5,15,17^. Antigen-specific IgA Abs neutralise viruses or toxins and interfere with the ability of pathogens to adhere to or penetrate through the mucosal epithelial barrier. Thus oral administration of probiotic LAB strains accelerates the clearance of viruses by promoting the production of virus-specific IgA at mucosal sites. In addition to antigen/pathogen-specific targeting by immunoglobulin, secretory IgA is equipped with glycan-dependent innate immunity, which protects the gut from pathogen invasion by inhibiting the adherence of diverse and variable mucosal microorganisms, *i.e*. their glycan-moieties compete with epithelial receptors for pathogens^18^. Given that both Ag-specific and non-specific IgA protect mucosal surfaces from pathogen invasion and colonization, the probiotic effect of orally administered LAB to enhance IgA production contributes to repelling pathogens^5,17^.

How intestinal IgA production is maintained under steady-state conditions has been intensively studied. As evidenced by the much lower levels of IgA produced in the guts of germ-free mice, commensal bacteria play a critical role^19^. Follicular helper T cells (Tfh) in small intestinal Peyer’s patches (PPs) that express Bcl6, PD-1, ICOS, and CXCR5 are critical cell populations for inducing IgA class-switch recombination and somatic hypermutation in germinal center B cells to produce high-affinity IgA, and this requires luminal innate immune signals^20^. There is also evidence that most of the IgA Abs induced by Tfh cells are specific for microbiota antigens, regulating the composition of mucosa-associated microbiota^21^. Furthermore, one indigenous opportunistic bacterial genus, *Alcaligenes*, was reported to inhabit PPs and induce local antigen-specific Ab production^22^. Thus, intestinal bacteria are involved in IgA production and are thought to regulate gut microbiota composition via communication with intestinal immune cells. To this end, IgA production is required for maintaining intestinal homeostasis under steady-state conditions, in addition to its obligatory task of repelling pathogens^23,24^.

Although how the positive and negative effects of IgA act to regulate the selection of intestinal microorganisms is obscure at present, it is clear that an abundance of IgA is required for a healthy gut environment and protective immune homeostasis^25^. We recently clarified that IFN-β is secreted from mDCs, uniquely, in response to LAB; we also observed that double-stranded RNA (dsRNA) in LAB was the main active component for stimulating endosomal TLRs^14,26^. In the present study, we investigated if this innate pathway is also important to IgA production from B cells, using human peripheral blood mononuclear cells (PBMCs). As being major luminal commensal bacteria in the small intestine and common components of fermented foods in human diet, LAB indeed enhanced IgA production from B cells, and importantly, via their RNA. Furthermore, for IgA production in human system IL-10 is as important as IL-6, which has been reported in murine experimental system^17^.

## Results

### IgA secretion by PBMCs in response to LAB via IL-6 and IL-10

**Figure 1:**
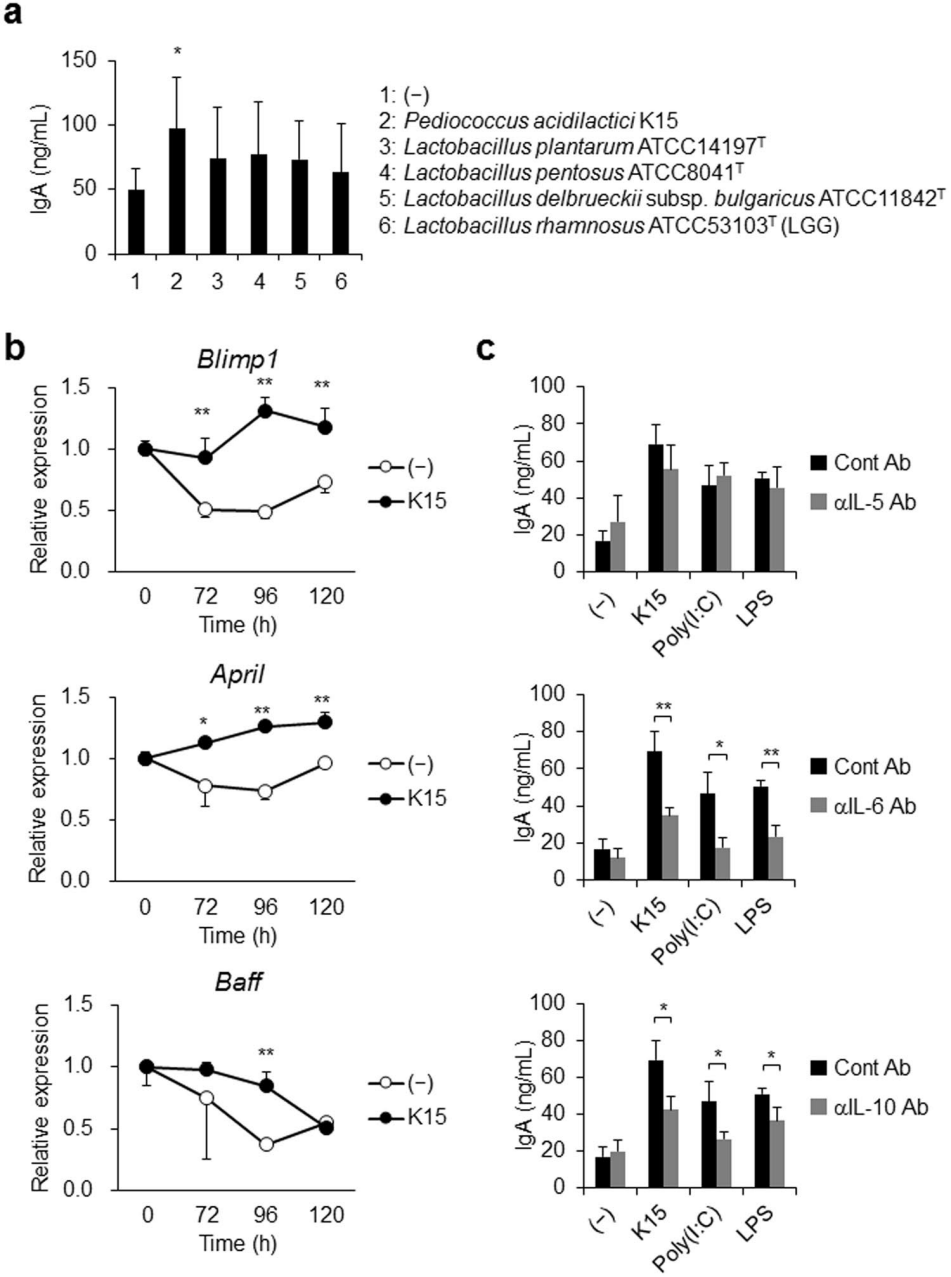
IgA secretion from peripheral blood mononuclear cells (PBMCs) in response to lactic acid bacteria (LAB) (**a**) PBMCs from 7 donors were cultured in medium alone (−) or stimulated with various strains of heat-killed LAB in triplicates for 5 days. The tested strains of LAB are described in Table S1. The resulting IgA concentrations were measured by ELISA. Data are represented as mean ± SD of 7 donors. *p < 0.05 (vs medium alone, Student’s *t*-test). (**b**) PBMCs were cultured in medium alone (−) or simulated with heat-killed K15. The resulting mRNA expressions of *Blimp-1*, *April*, and *Baff* were measured by qPCR. Data are represented as mean ± SD of duplicates and are representative of two independent experiments from different individuals. *p < 0.05, **p < 0.01 (Student’s *t*-test). (**c**) PBMCs were cultured in medium alone (−) or stimulated with heat-killed K15, poly(I:C) (10 μg/mL), or LPS (10 μg/mL) for 5 days in the presence of control Ab (cont Ab), anti-IL-5 mAb (αIL-5 Ab), anti-IL-6 mAb (αIL-6 Ab), or anti-IL-10 mAb (αIL-10 Ab). The resulting IgA concentrations were measured by ELISA. Data are represented as the mean ± SD of triplicates and are representative of two independent experiments from different individuals. *p < 0.05, **p < 0.01 (Student’s *t*-test).

We evaluated the IgA secretion by PBMCs from 7 donors in response to some strains of LAB (described in Table S1). Among tested strains of LAB, *Pediococcus acidilactici* K15 significantly induced IgA production in PBMCs (Fig. 1a). The other strains of LAB failed to enhance IgA production in this experimental system (Fig. 1a). B lymphocyte-induced maturation protein 1 (Blimp-1) is a master regulator for plasma cell differentiation^27^, and B cell activating factor belonging to the TNF family (BAFF) and a proliferation inducing ligand (APRIL) are both known to promote this class switching^28–30^. In K15-stimulated PBMCs, the gene expression encoding Blimp-1 and APRIL was upregulated, whereas that encoding BAFF did not change, in comparison with unstimulated PBMCs (Fig. 1b). Reportedly, IgA secretion by B cells is activated by IL-5 or IL-6 in mouse PBMCs^17,31^ and by IL-6 and IL-10 in human PBMCs^32^. These cytokines promote the differentiation of IgA-producing plasma cells. We performed experiments in the presence of neutralizing Abs against IL-5, IL-6, and IL-10 to clarify which factor is involved in IgA production by human PBMCs in response to K15. Although a neutralizing Ab against IL-5 did not affect IgA secretion in response to K15 or to TLR ligands, both neutralizing Abs against IL-6 and IL-10 impaired IgA induction by their respective cytokines (Fig. 1c). As IL-5 was not involved in the induction of IgA secretion, the induction of IgA by LAB-stimulation likely occurs via a T cell-independent pathway. These results indicate that, in human PBMCs, the effects of LAB on activating IgA production are induced by IL-6 and IL-10, most likely secreted by DCs in response to LAB.

### IgA production by B cells via the DC response to LAB

**Figure 2:**
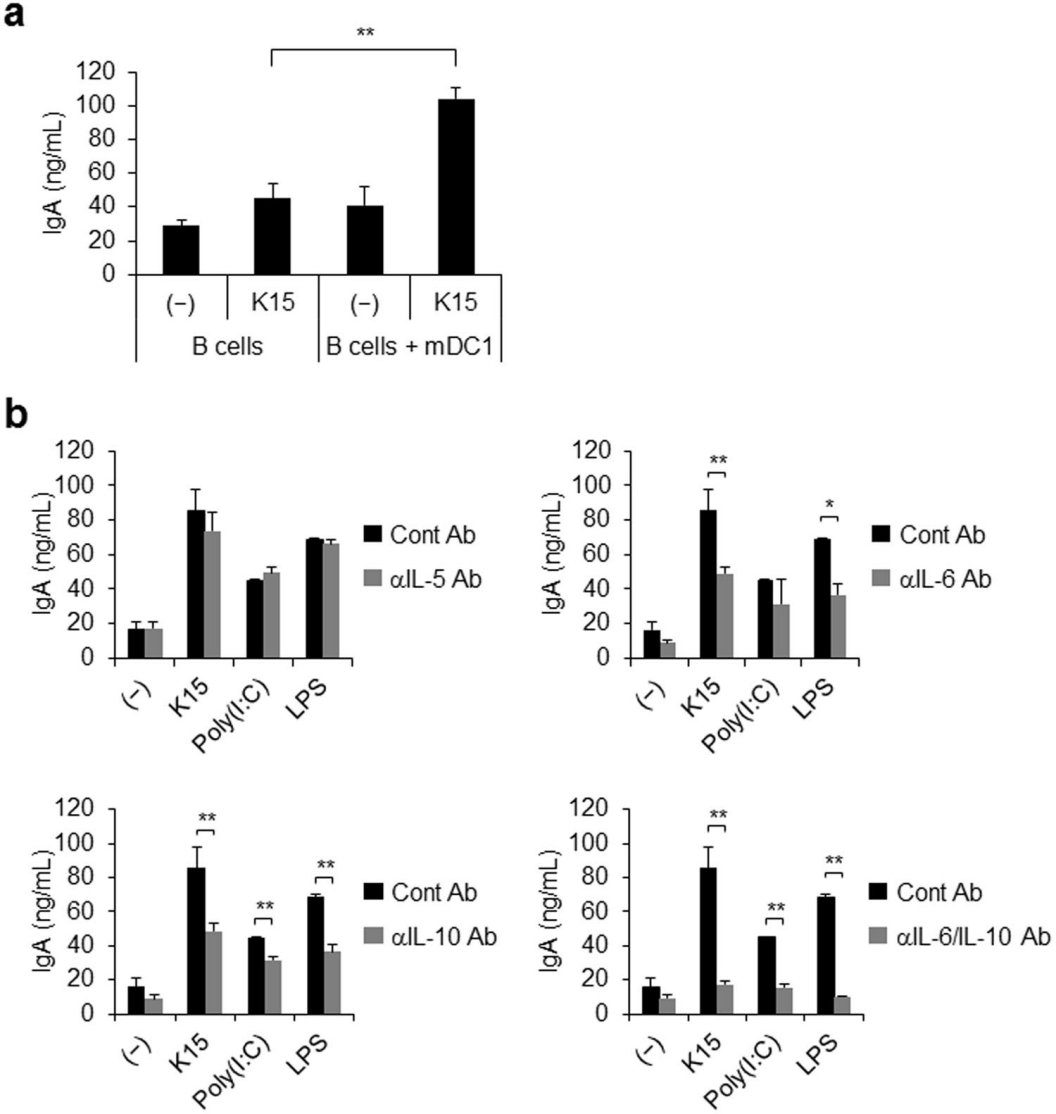
IgA secretion from B cells in the presence of dendritic cells (DCs) and/or LAB (**a**) B cells and BDCA1^+^ DCs (mDC1s) were isolated from PBMCs. B cells were co-cultured with heat-killed K15 in the presence or absence of mDC1s for 5 days. The resulting IgA concentrations were measured by ELISA. (**b**) B cells and mDC1s were cultured with K15, poly(I:C) (10 μg/mL), or LPS (10 μg/mL) for 5 days in the presence of control Ab (cont Ab), anti-IL-5 mAb (αIL-5 Ab), anti-IL-6 mAb (αIL-6 Ab), and/or anti-IL-10 mAb (αIL-10 Ab). The resulting IgA concentrations were measured by ELISA. (**a**,**b**) Data are represented as the mean ± SD of triplicates and are representative of two independent experiments from different individuals. *p < 0.05, **p < 0.01 (Student’s *t*-test).

LAB contain many kinds of TLR ligands, such as bacterial cell walls, which are ligands for TLR2 and TLR4, dsRNA, which is a ligand of TLR3, and DNA, which is a ligand of TLR9^14,33–36^. DCs are activated by these TLR ligands, and these cells, once activated, secrete many kinds of cytokines and IFNs. We isolated BDCA1^+^ DCs (mDC1s) and B cells from PBMCs and co-cultured them in the presence of neutralizing Abs against IL-5, IL-6, and IL-10. Following stimulation by K15, IgA production by B cells was slightly activated in the absence of mDC1s, whereas it was strongly activated in the presence of mDC1s (Fig. 2a). This indicates that DCs contribute to the activation of IgA secretion in response to LAB. When neutralizing Abs against IL-5, IL-6, and IL-10 were added to this co-culture system, the Abs against IL-6 and IL-10 significantly suppressed IgA production in response to LAB, and the combination of both of these Abs completely abolished IgA production (Fig. 2b). This result is in accordance with the observed IgA production by PBMCs described above (Fig. 1b). Together, these results strongly suggest that the IL-6 and IL-10 secreted by DCs in response to LAB activate IgA production by B cells. Previous reports identified IL-6 as a critical factor for IgA induction by LAB in mice^17^. However, our present research clearly demonstrates that IL-10 also makes a major contribution to this process in humans.

### IL-6 and IL-10 secretion by DCs in response to bacterial RNA

**Figure 3:**
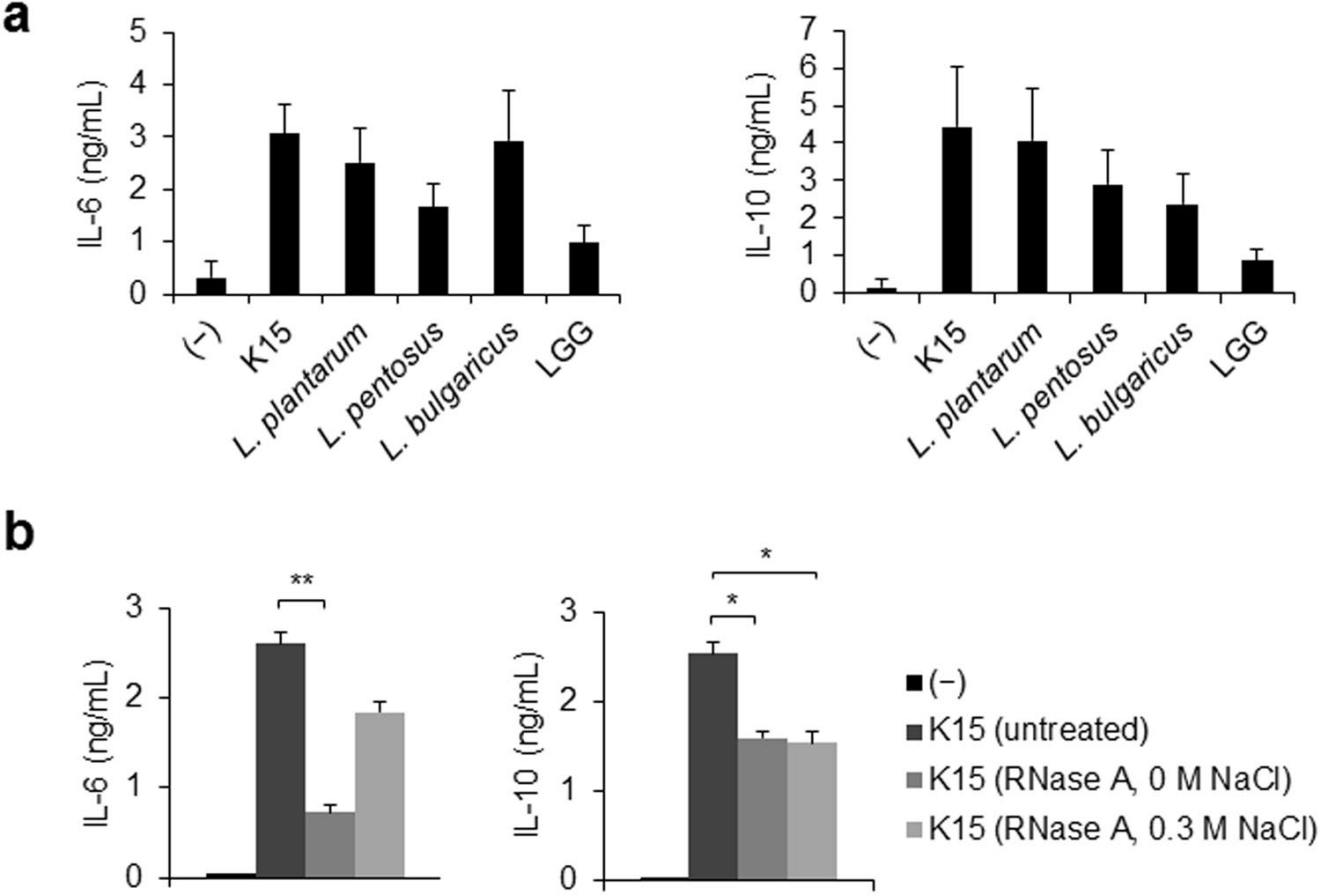
IL-6 and IL-10 production by mDC1s in response to LAB (**a**) mDC1s were isolated from PBMCs of 7 donors. mDC1s were stimulated with heat-killed LAB in duplicates for 24 h. The tested strains LAB and their abbreviations are described in Table S1. The resulting IL-6 and IL-10 concentrations were measured by ELISA. Data are represented as mean ± SD of 7 donors. (**b**) Heat-killed K15 cells were treated with RNase A under 0 M NaCl for digestion of ssRNA and dsRNA or under 0.3 M NaCl for digestion of ssRNA only. mDC1s were cultured with heat-killed K15 or RNase A-treated, heat-killed K15 for 24 h. The resulting IL-6 and IL-10 concentrations were measured by ELISA. Data are represented as the mean ± SD of duplicates and are representative of two independent experiments from different individuals. *p < 0.05, **p < 0.01 (Student’s *t*-test).

After finding that IL-6 and IL-10 are involved in the activation of IgA production that is induced by LAB, we investigated the levels of these cytokines secreted by mDC1s in response to several strains of LAB. K15 activated IL-6 and IL-10 production by DCs, and *L. bulgaricus* ATCC11842^T^ strongly induced IL-6 production (Fig. 3a). We previously reported that LAB contain a larger amount of dsRNA than pathogenic bacteria^14^. To test if RNA in LAB is responsible for the activation of IL-6 and IL-10 production by mDC1s, and thus in inducing IgA secretion, heat-killed K15 were treated with RNase A under 0 M NaCl to degrade both ssRNA and dsRNA or under 0.3 M NaCl to degrade only ssRNA^37^. IL-6 production by mDC1s in response to K15 was strongly impaired by the degradation of both ssRNA and dsRNA (Fig. 3b) but not significantly by the degradation of ssRNA alone, indicating that bacterial dsRNA is the major component of LAB that induces IL-6 production. In contrast, IL-10 production was partially impaired by degradation under both conditions (Fig. 3b), indicating that ssRNA, but not dsRNA, contributes to IL-10 induction.

### Activation of salivary sIgA production by K15 ingestion in a clinical trial

The effect of LAB on salivary IgA production was evaluated in a randomized, placebo-controlled trial using heat-killed K15. The secretion rate of salivary sIgA was significantly increased by ingestion of K15 for 8 and 12 weeks compared with baseline (*p* = 0.021 for 8 weeks, *p* = 0.002 for 12 weeks) and samples from participants who had ingested a placebo for 12 weeks (*p* = 0.036) (Table 1). The salivary sIgA concentration was significantly enhanced only in the K15 group (*p* = 0.003) (Table 1). These results indicate that K15 ingestion enhances salivary sIgA production, likely via IL-6 and IL-10 secreted by DCs, resulting in the activation of anti-viral or anti-bacterial immune responses. Any adverse reactions were not observed.

**Table 1:** Salivary sIgA secretion rate, saliva flow rate, and salivary sIgA concentrations in placebo group (n = 25) and K15 group (n = 27).

Group	Before	4 weeks	8 weeks	12 weeks
Salivary sIgA secretion rate (μg/min)				
Placebo	160.6 ± 8.3	189.7 ± 18.3	205.3 ± 24.2	211.7 ± 21.9^#^
K15	148.6 ± 8.8	162.1 ± 18.9	195.8 ± 21.6^#^	208.9 ± 20.2^##^
Saliva flow rate (g/min)				
Placebo	0.619 ± 0.042	0.660 ± 0.034^#^	0.655 ± 0.035	0.653 ± 0.027
K15	0.623 ± 0.046	0.599 ± 0.044	0.646 ± 0.040	0.616 ± 0.041
Salivary sIgA concentration (mg/dL)				
Placebo	31.5 ± 4.3	30.9 ± 3.6	32.5 ± 4.3	34.1 ± 4.2
K15	27.2 ± 2.7	28.4 ± 2.9	31.6 ± 3.6	37.4 ± 4.1^##^

mean ± SE, ^#^*p* < 0.05, ^##^*p* < 0.01 compared with baseline according to a Student’s *t*-test.

## Discussion

It has been reported that probiotic strains of LAB activate IgA production by B cells; this molecular mechanism was revealed in mice^17^. In the present study, we elucidated the mechanism for the effect of a functional LAB strain, *Pediococcus acidilactici* K15, on IgA production, focusing on the role of bacterial RNA in the interaction between DCs and B cells from human peripheral blood. In fact, K15, which activated IgA secretion from PBMCs *in vitro*, upregulated salivary sIgA concentration in healthy adults.

In mice, IL-6 secretion by PP DCs in response to some strains of *Lactobacillus* sp. promoted IgA^+^ B cells to differentiate into IgA-producing plasma cells^17^. The present study found that in human PBMCs, IL-10 is another critical factor for activating IgA production from B cells. Moreover, we demonstrated via the use of a co-culture system composed of PBMC-derived mDC1s and B cells that the activation of IgA production by K15 is dependent on the function of DCs and their recognition of bacterial RNA.

A number of studies using animal models have revealed the beneficial effects of orally administered LAB, including *Pedioccocus acidilactici*^5,6,14,17,38^, suggesting that orally administered LAB are recognized in the intestine by antigen-presenting cells including DCs and macrophages. In our clinical trial, we confirmed that the salivary sIgA concentration was enhanced by K15 ingestion. Although we did not directly analyse the DCs in the guts of our human volunteers, our *in vitro* data and previous work on the immune events in the intestine suggest that the ingested K15 were probably recognized by DCs in PPs or the lamina propria, which then upregulated the IgA concentration in saliva. Arase *et al*. reported that a strong correlation is observed in humans between the levels of challenge-bacteria-specific IgA Ab in sublingual/submandibular secretions and those in gut lavages^39^.

For augmentation of sIgA production at mucosal sites, an IgA class switch recombination to IgA^+^ B cells induced by APRIL and BAFF is necessary^28–30^. In humans, it has been reported that APRIL, but not BAFF, is produced by monocyte-derived DCs following stimulation with CpG DNA or poly(I:C), although other TLR ligands were not able to induce either molecule^40^. In mice, all-trans-retinoic acid (RA) and IL-4 were shown to promote IgA class switching in cooperation with IL-5^41^, but we found that this mechanism was not involved in the IgA induction in our culture system. Here, we observed that the mRNA expression levels for APRIL, BAFF, and Blimp-1 were increased in PBMCs in the presence of LAB, which suggests that LAB may be sufficient to activate B cells for class switching and may also directly activate Tfh in the intestine. We also demonstrated in the present study that LAB RNA is the active component inducing the cytokine production that is critical for the enhancement of IgA production by B cells; dsRNA is a major causative molecule for IL-6 production whereas single-stranded RNA is critical factor for IL-10 production. Further investigations are needed to determine if these bacterial ligands to TLRs also contribute to IgA class switching and the promotion of IgA production via DC and Tfh functions.

Here, we used mDC1s isolated from PBMCs in a co-culture system with B cells. In addition to mDC1s, BDCA3^+^ DCs (mDC2s) and pDCs are also part of the composition of PBMCs, and, among these DC subsets, mDC1s and pDCs are the more abundant cell populations^42,43^. mDC2s express a high level of TLR3, which recognizes the dsRNA in viruses and bacteria and secretes IFN-λ, a type III IFN^44^. pDCs express TLR7/8 and TLR9 and robustly secrete IFN-α during viral infections^16,45–47^. mDC1s expressing many kinds of TLRs have been found to secrete IFN-β and many kinds of cytokines, including IL-12 and IL-27, in response to poly(I:C), R848, and LPS^43,48–50^. Additionally, mDC1s secrete higher levels of IL-6 in response to *Staphylococcus aureus*, whose active ligand is TLR4^48^. Furthermore, IL-10 and IL-6 secretion levels in response to poly(I:C) were also reported to be higher in mDC1s than in mDC2s^51^. Thus, mDC1s seem to make a greater contribution to immunity than other DC subsets in the case of IgA induction via IL-6 and IL-10 production upon stimulation with bacteria.

In addition to IgA induction, probiotic bacteria aid a variety of aspects relating to anti-infection immune functions. We previously reported that LAB contain large amounts of dsRNA, which induces IFN-β production via the TLR3 signalling pathway^14^. Type I IFNs, IFN-α and IFN-β, are well known to trigger a robust immune response against viral and bacterial infections^13,52^. LAB also activate IL-12 secretion from DCs or macrophages, which promotes Th1 cell differentiation, natural killer cell activation, and cytotoxic CD8^+^ T cell activation in concert with IL-27 and IL-18 production^49,53^. These subsets of cells protect against viral infection as well. Together, previous work and our current study suggest that functional strains of LAB, which has high safety for human and induce the production of IFN-β, IL-12, IL-6, and IL-10 by mDCs, protect the human body against viral and bacterial infection and maintain immune homeostasis by augmenting many of the pathways involved in protective immune responses.

## Methods

### Preparation of LAB for *in vitro* experiments

LAB were purchased from the Japan Collection of Microorganisms (JCM) or isolated from fermented foods (Table S1). *Pediococcus acidilactici* K15, *Lactobacillus plantarum* ATCC14197^T^, and *Lactobacillus pentosus* ATCC8041^T^ were cultured at 30 °C for 24 h in MRS broth (Becton Dickinson, Sparks, MD, USA). *Lactobacillus delbrueckii* sup. *bulgaricu*s ATCC11842^T^ and *Lactobacillus rahmnosus* ATCC53103^T^ (LGG) were cultured at 37 °C for 24 h in MRS broth. They were then heat-killed, washed twice with saline, and suspended in saline. For nuclease treatment of the heat-killed bacteria, treatment with RNase A from bovine pancreas (Sigma, St. Louis, MO, USA) was performed under low salt conditions (10 mM Tris-HCl, pH 8.0) or high salt conditions (10 mM Tris-HCl, 0.3 M NaCl, pH 8.0) at 37 °C for 2 h. RNase A-treated bacteria were washed twice with each buffer and used for subsequent experiments.

### Preparation of PBMCs, B cells, and mDC1s

These *in vitro* experiments were carried out in accordance with the Ethics Committee of Kikkoman Corporation (Chiba, Japan, No. KC-RD9), and blood samples were acquired from healthy volunteers under informed written consent in compliance with the Declaration of Helsinki (2013). PBMCs were isolated by using Ficoll-Paque PLUS (GE Healthcare, Piscataway, NJ, USA). B cells were isolated from PBMCs by using CD19 microbead labelling and the MACS system (Miltenyi Biotec, Bergisch-Gladbach, Germany). Cell purity was typically over >95% as assessed by staining with FITC-conjugated anti-CD19 Ab (Biolegend, San Diego, CA, USA). mDC1s were isolated by using a CD1c (BDCA1)^+^ Dendritic Cell Isolation kit (Miltenyi Biotec) according to the manufacturer’s instructions. Cell purity was >98% as assessed by staining with FITC-conjugated anti-CD11c, BV421-conjugated anti-CD1c, and APC-conjugated HLA-DR.

### Cell culture

Cell culture assays were performed in RPMI 1640 (Gibco, Paisley, UK) including 10% heat-inactivated fetal bovine serum (HyClone Laboratories, Logan, Utah), MEM Vitamin Solution (100×, Gibco), MEM Non-Essential Amino Acids (100×, Gibco), Penicillin Streptomycin (100×, Gibco), Sodium Pyruvate (100 mM, Gibco) and 2-Mercaptoethanol (1000×, Gibco) in a humidified incubator (5% CO_2_) at 37 °C. PBMCs were cultured in 96-well round-bottomed plates at 2 × 10^5^ cells/well/250 μl in the presence or absence of 1 × 10^7^ bacteria for 5 days. B cells were cultured at 4 × 10^4^ cells/well/250 μl with 2 × 10^4^ mDC1s in the presence or absence of 1 × 10^7^ bacteria for 5 days. The level of IgA in culture supernatants was measured by ELISA using anti-human IgA mAb and biotin-conjugated anti-human IgA mAb (BD Pharmingen, San Diego, CA, USA). mDC1s were cultured at 5 × 10^4^ cells/well/200 μl with 1 × 10^7^ bacteria, and the levels of IL-10 and IL-6 in culture supernatants were determined by corresponding ELISA kits (eBiosciences, San Diego, CA, USA).

### Reagents

To neutralize IL-5, IL-6, and IL-10, monoclonal antibody (mAb) against each cytokine (Biolegend) was added at 10 μg/ml. Rat IgG_1_ Ab (Biolegend) was used as an isotype control Ab. Poly(I:C) and LPS (both purchased from Invivogen, San Diego, CA, USA) were added at 10 μg/ml as representative TLR3 and TLR4 ligands, respectively.

### Quantitative RT-PCR

Total RNA was extracted from the cells with a NucleoSpin RNA Kit (Takara, Kyoto, Japan) following the manufacturer’s instructions. An equal amount of total RNA (300 ng) corresponding to each priming dose was reverse-transcribed using PrimeScript RT Reagent (Takara). The cDNA obtained after reverse transcription was amplified using specific primers for Blimp-1, APRIL, BAFF, and β-actin purchased from Takara and SYBR Premix Ex Taq II (Takara) following the protocols provided. The analysis of each gene expression was performed by normalizing to β-actin.

### Preparation of clinical test foods

Test foods were prepared by Kikkoman Corporation. K15 was cultured in medium including soy peptone, yeast extract, and glucose. The cultured K15 were then heat-killed, washed with saline, concentrated, and spray-dried with dextrin. The test foods for the K15 and placebo groups were prepared using this K15 powder and a dextrin powder (Table S2). The subjects in the K15 group ingested 1 g of dextrin including 9.1 mg of heat-killed K15 (5 × 10^10^ bacteria) every day for 12 weeks, while the subjects in the placebo group ingested 1 g of dextrin alone. Bacterial number in K15 samples was counted by using Nikon Eclipse 80i microscope (400×, Japna) and Micro Slide Glass (Matsunami Glass Ind., Ltd, Japan). The participants and the researchers were not able to discriminate between placebo and K15 samples.

### Clinical study design

This study took place at Ueno Clinic, Aisei Hospital (Tokyo, Japan) between June 2016 and March 2017. A randomized, double-blinded, placebo-controlled trial was performed for 12 weeks. The trial was conducted by TTC Co. Ltd. funded by Kikkoman Corporation and approved by the Ethics Committee of Ueno Clinic, Aisei Hospital (Protocol No. 20160609–2) in compliance with the Declaration of Helsinki (2013). The trial was registered in the UMIN Clinical Trials Registry as UMIN000022880 (date of registration 01/04/2017). All participants provided written informed consent approved by the ethics committee. Data for body height, body weight, BMI, blood pressure, pulse, and biochemistry tests (BML Co. Ltd., Saitama, Japan) were obtained for all subjects before treatment and every 4 weeks after treatment. All subjects were followed up until the physician confirmed they had no important harms, and any important harms related to test foods were not observed. All subjects answered Profile of Mood States 2 Adult Short Form (POMS 2-A) and questionnaires about their weekly life events to confirm their fatigue and psychological stress. The salivary sIgA secretion rate was the primary outcome, and the salivary sIgA concentration and T scores in POMS 2-A were the secondary outcomes. We confirmed that Fatigue–Inertia (FI) T scores and Vigor–Activity (VA) T scores were significantly improved in both groups and were not different between two groups (Table S3).

### Subjects for a clinical trial

The subjects were recruited from volunteers enrolled in Medical Art Laboratory Co. Ltd. (Tokyo, Japan), and 60 subjects were selected from 382 volunteers by the selection and exclusion criteria. The selection criteria were as follows: subjects who (a) were 20–64 years old and healthy; (b) had a lower than average salivary sIgA secretion rate; and (c) had a FI T score of more than 50 with a VA T score of less than 50 as assessed by POMS 2-A. The exclusion criteria were follows: subjects who (a) consumed food containing lactic acid bacteria, such as yogurt and other fermented foods; (b) had an allergic disease; (c) had mouth or teeth issues or were under treatment of them; (d) routinely engaged in vigorous exercises; (e) were pregnant or lactating; or (f) were inappropriate cases for the trial as defined by physicians. The researcher independent of this study (TTC Co. Ltd.) performed simple randomized allocation of the subjects by using a computer-generated list of random numbers. The allocation sequence was concealed from the researcher enrolling and assessing participants in the sealed envelope. This allocation sequence was opened by the independent researcher described above only after the enrolled participants completed all assessments.

### Determination of salivary sIgA secretion rate and concentration

Saliva samples were collected for measurement of sIgA concentration and saliva flow rate before and every 4 weeks after the start of K15 or placebo ingestion. Salivary sIgA concentration was determined by ELISAs performed by Daiichi Kishimoto Clinical Laboratories, Inc. (Hokkaido, Japan). The salivary sIgA secretion rate was calculated from the saliva flow rate and salivary sIgA concentration.

### Statistical analysis

Error bars on figures indicate the standard deviation (SD) of duplicate or triplicate samples for cell culture assay experiments. Statistical analysis was performed by PASW Statistics Version 18.0 (SPSS Inc, Chicago, IL, USA). The statistical significance was determined with a two-tailed Student’s *t*-test for unpaired data, with *p* values of <0.05 considered significant (indicated on figures and tables as *p < 0.05, **p < 0.01). In the clinical trial, the consumption of test samples was monitored via diaries kept by the subjects, and these reports indicated that all subjects consumed more than 90% of total test samples. Two subjects (placebo group: n = 1, K15 group: n = 1) were excluded from the analysis due to meeting the exclusion criteria during the test period (Fig. S1). The subjects whose saliva flow rate strongly changed during the test period (CV > 1.5 SD) were also excluded from the analysis (placebo group: n = 4, K15 group: n = 2) (Fig. S1). Error bars on the figures presenting these data indicate standard error (SE). The statistical significance between two groups was determined with a two-tailed Student’s *t*-test for unpaired data, with *p* values of <0.05 considered significant. The comparison between values from before and after the ingestion of test samples was performed by a paired *t*-test.

## Electronic supplementary material

Supplementary Information
