# Supplementary material for: The molecular mechanism for activating IgA production by Pediococcus acidilactici K15 and the clinical impact in a randomized trial
Source: Sci Rep. 2018 Mar 22;8:5065. doi: 10.1038/s41598-018-23404-4 (PMC5864838; doi:10.1038/s41598-018-23404-4)
Supplement: Supplementary file 1 — Supplementary Information [file 41598_2018_23404_MOESM1_ESM.docx]

Supplementary Information

The molecular mechanism for activating IgA production by *Pediococcus acidilactici* K15 and the clinical impact in a randomized trial

Tadaomi Kawashima^a,b,*^, Naho Ikari^a,b^, Tomoko Kouchi^a^, Yasuyuki Kowatari^c^, Yoshiro Kubota^d^, Naoki Shimojo^e^ and Noriko M Tsuji^b^

^a^ Research and Development Division, Kikkoman Corporation, Chiba, Japan

^b^ Biomedical Research Institute, National Institute for Advanced Industrial Science and Technology (AIST), Tsukuba, Japan

^c^ Ueno Clinic, Aisei Hospital, Tokyo, Japan

^d^ Kikkoman General Hospital, Kikkoman Corporation, Chiba, Japan

^e^ Department of Pediatrics, Graduate School of Medicine, Chiba University, Chiba, Japan

* Corresponding author.

Correspondence and requests for materials should be addressed to T. Kawashima (email: takawashima@mail.kikkoman.co.jp).

Supplementary Tables

Table S1 Strain numbers and organism names of the lactic acid bacteria used in this study

| Organism name | Strain No. | Abbreviation |
| --- | --- | --- |
| *Pediococcus acidilactici* | K15 | K15 |
| *Lactobacillus plantarum* | ATCC14197^T^ | *L. plantarum* |
| *Lactobacillus pentosus* | ATCC8041^T^ | *L. pentosus* |
| *Lactobacillus delbrueckii* subsp. *bulgaricu*s | ATCC11842^T^ | *L. bulgaricus* |
| *Lactobacillus rhamnosus* | ATCC53103^T^ | LGG |

Table S2 Analysis of test food

| Nutrition (per g powder) | Placebo | K15 powder |
| --- | --- | --- |
| Energy (kcal) | 3.84 | 3.83 |
| Protein (g) | 0.00 | 0.01 |
| Lipid (g) | 0.00 | 0.00 |
| Carbohydrate (g) | 0.96 | 0.95 |
| Sodium (mg) | 0.01 | 0.12 |
| Heat-killed K15 (mg) | 0.00 | 9.10 |

Table S3 Results of Fatigue-Inertia (F-I) and Vigor-Activity (V-A) T scores in POMS 2-A in placebo group (n=25) and K15 group (n=27).

|  | Group | Before | 4 weeks | 8 weeks | 12 weeks |
| --- | --- | --- | --- | --- | --- |
| FI | Placebo | 65.0 ± 1.6 | 56.6 ± 1.5^##^ | 53.5 ± 2.0^##^ | 51.6 ± 1.7^##^ |
|  | K15 | 63.5 ± 1.5 | 57.1 ± 2.5^##^ | 54.1 ± 2.4^##^ | 52.9 ± 2.9^##^ |
|  | t-test | n. s. | n. s. | n. s. | n. s. |
| VA | Placebo | 40.1 ± 1.1 | 45.9 ± 1.9^##^ | 45.2 ± 2.0^##^ | 45.4 ± 1.8^##^ |
|  | K15 | 40.6 ± 1.0 | 45.2 ± 1.7^##^ | 46.8 ± 1.8^##^ | 46.2 ± 2.1^##^ |
|  | t-test | n. s. | n. s. | n. s. | n. s. |

mean ± SE, ^#^*p*<0.05, ^##^*p*<0.01 compared with baseline based on a Student’s *t*-test

Supplementary Figure

**Figure S1 Study flow and number of subjects in each phase of the clinical trial.**
